# Phycoerythrin Peptide from *Pyropia yezoensis* Alleviates Endoplasmic Reticulum Stress Caused by Perfluorooctane Sulfonate-Induced Calcium Dysregulation

**DOI:** 10.3390/md16020044

**Published:** 2018-01-26

**Authors:** Jeong Hwan Oh, Eun-Young Kim, Taek-Jeong Nam

**Affiliations:** Institute of Fisheries Sciences, Pukyong National University, Busan 46041, Korea; ojhwan55@pknu.ac.kr (J.H.O.); letyoung@naver.com (E.-Y.K.)

**Keywords:** persistent organic pollutant, perfluorooctane sulfonate, endoplasmic reticulum stress, phycoerythrin-derived peptide, *Pyropia yezoensis*

## Abstract

Perfluorooctane sulfonate (PFOS), a stable fluorosurfactant, causes endoplasmic reticulum (ER) stress in the brain. This study was designed to investigate whether a phycoerythrin-derived peptide of *Pyropia yezoensis* (PYP) reduces PFOS-induced ER stress associated with calcium dysregulation. The protective effects of PYP were determined by cell viability, immunoblotting for ER stress response protein glucose-regulated protein 78 (GRP78) and calcium-dependent protein kinases in rat frontal cortical neurons. PFOS-induced decrease in cell viability was attenuated by PYP pretreatment (1 µg/mL) for 24 h, which was downregulated by inhibiting tropomyosin-receptor kinase B (TrkB). PYP pretreatment downregulated the increase in intracellular calcium levels and phosphorylation of calcium/calmodulin-dependent protein kinase II and c-Jun N-terminal kinase which are associated with a PFOS-induced increase in GRP78. The PFOS-induced increase in GRP78 was downregulated via activation of TrkB receptor-linked extracellular signal-regulated kinases 1/2 (ERK1/2) by PYP pretreatment. Moreover, PYP microinjections (1 µg/kg, 0.54 nmol) attenuated the GRP78 expression in rat prefrontal cortex caused by PFOS (10 mg/kg) exposure for 2 weeks. These findings demonstrate that PYP enhances frontal cortical neuron viability via activation of TrkB receptor-ERK1/2 signaling and attenuation of ER stress in rat prefrontal cortex against PFOS exposure, suggesting that PYP might prevent neuronal dysfunctions caused by PFOS-induced ER stress.

## 1. Introduction

The synthetic fluorosurfactant, perfluorooctane sulfonate (PFOS), has been used to protect the surfaces of papers, food containers, carpets, and a variety of other surfaces owing to its hydrophobic and lipophobic properties [1]. Due to their strong carbon-fluorine (C-F) covalent bond, synthetic fluorosurfactants have a relatively long depuration half-life and have been considered as persistent organic pollutants [2,3], which suggests that PFOS accumulates in tissues including the brain through food chains, and causes adverse effects on humans, such as breast cancer and reproductive dysfunction [4,5,6]. PFOS could also induce oxidative stress and cellular damage such as hepatocellular hypertrophy [7], inhibit intracellular communication [8], and cause developmental toxicity in mice [9,10]. Recent studies have also demonstrated that PFOS could cause brain dysfunction, including neurobehavioral defects, cognitive impairments, and immunotoxicity by crossing the blood-brain barrier [11,12,13,14,15,16].

The endoplasmic reticulum (ER) plays a critical role in protein modification and folding as well as intracellular calcium homeostasis. Cellular stress-induced protein damage and alteration of the redox status can cause a reduction of folding capacity and accumulation of misfolded proteins in the ER lumen, thereby activating a series of signaling pathways known as the ER stress response [17,18]. The 78-kDa glucose-regulated protein (GRP78), an ER stress sensor, is an adenosine triphosphate-dependent protein chaperone for protein quality control localized in the ER lumen. Upon ER stress, GRP78 binds unfolded proteins and activates a multi-chaperon complex, resulting in increased ER protein folding capacity [19]. Calcium dysregulation and long-lasting ER stress can lead to the accumulation of unfolded or misfolded proteins, subsequently leading to cell death. Collectively, the accumulated PFOS in the brain could adversely affect normal ER function because PFOS increases oxidative stress by dysregulating calcium levels in the neurons, possibly inducing cognitive impairment and neurodegenerative diseases [14,20].

A red alga, *Pyropia yezoensis* (*P. yezoensis*), is widely cultured as food and as a nutritional supplement for its biofunctional components such as proteins, vitamins, minerals, and mycosporine-like amino acids [21,22]. Moreover, peptides or glycoproteins derived from *P. yezoensis* protect against oxidative stress induced by acetaminophen or hydrogen peroxide in Chang and human hepatoblastoma HepG2 cells [23,24], and promote cell proliferation in intestinal epithelial IEC-6 cells via the activation of phosphatidylinositol 3-kinase (PI3K)-Akt signaling [25]. A recent study also demonstrated that a peptide derived from *P. yezoensis* (PYP) regulated muscle atrophy by inhibiting atrogin1/muscle atrophy F-box (MAFbx) and muscle RING Finger 1 (MuRF1) signaling in mouse myoblast C2C12 cells [26]. However, the effects of PYP in regulating ER stress in the brain remain unclear because most studies have focused on in vitro models that are irrelevant to the central nervous system.

Therefore, in this study, we hypothesized that PYP treatment regulated PFOS-induced ER stress and calcium dysregulation in rat prefrontal cortex. To verify this hypothesis, the study investigated the association between the protective effect of PYP against PFOS exposure with regulation of GRP78 expression in frontal cortical neurons and in rat prefrontal cortex. Thus, we investigated whether: (1) PYP pretreatment downregulated a decrease in cell viability, and (2) PYP pretreatment attenuated the PFOS-mediated increase in GRP78 expression and intracellular calcium levels, and (3) which is associated with tropomyosin-receptor kinase B (TrkB)-linked signaling pathways.

## 2. Results

### 2.1. PYP Pretreatment Attenuated PFOS-Induced Decrease in Viability of Frontal Cortical Neurons and the PYP-Induced Enhancement of Cell Viability Was Downregulated by Inhibiting TrkB Receptor

**Figure 1:**
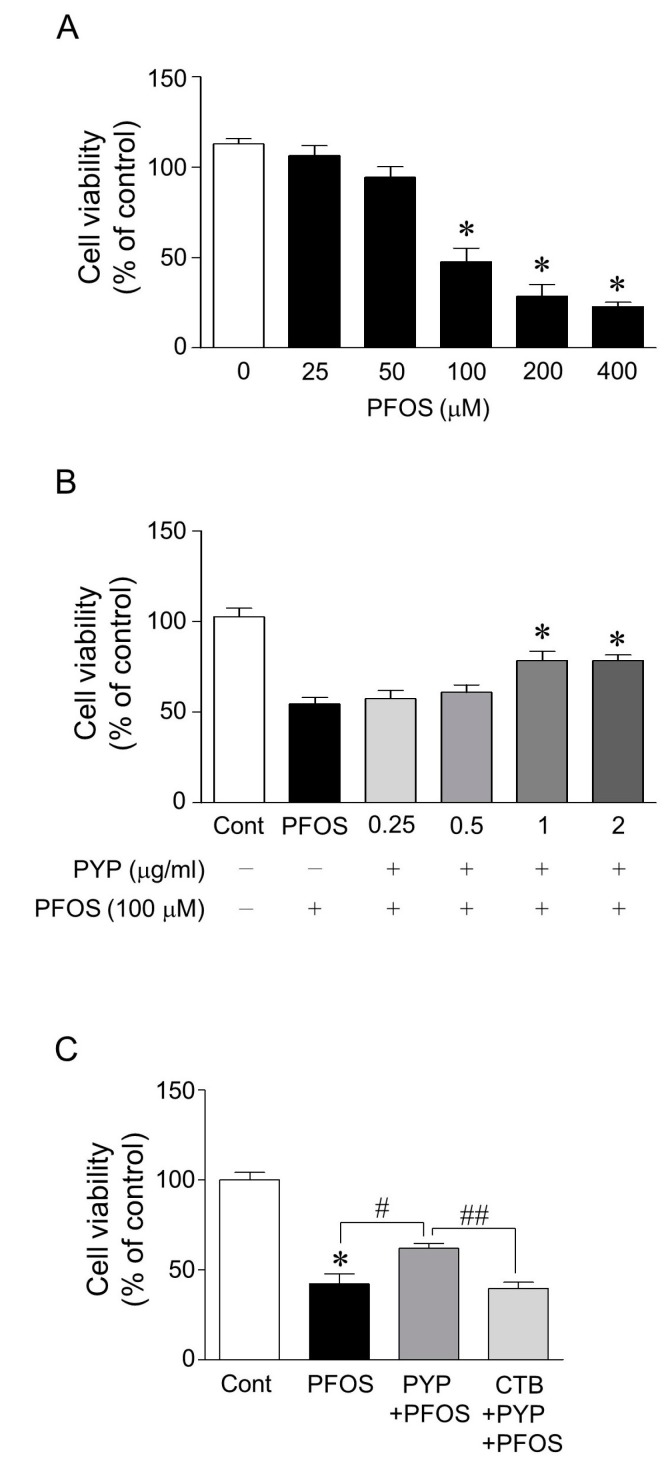
Cell viability of rat frontal cortical neurons following perfluorooctane sulfonate (PFOS) exposure with or without peptide derived from *P. yezoensis* (PYP) pretreatments. Exposure to PFOS (25–400 µM) for 24 h decreased the viability of rat frontal cortical neurons in a dose-dependent manner. A significant difference was observed from 100–400 µM of PFOS (**A**); Pretreatments with PYP (1–2 µg/mL) significantly downregulated PFOS-induced decrease in cell viability (**B**); which was abolished by inhibiting the TrkB receptor with 200 nM of cyclotraxin B (**C**). The data were expressed as the mean ± SEM of three independent experiments, each performed in triplicate. * *p* < 0.05 versus control group; ^#^ *p* < 0.05 versus PFOS treatment; ^##^ *p* < 0.05 versus PYP + PFOS treatments; CTB, cyclotraxin B; Cont, control.

To investigate the protective effects of PYP against PFOS-induced ER stress, the doses of PFOS and PYP to be used were first determined based on cell viability assay. Following PFOS exposure (25–400 µM) for 24 h, the viability of frontal cortical neurons (12–14 days in vitro) significantly decreased in a dose-dependent manner between 100 and 400 µM PFOS (Figure 1A). The cell viability decreased by half at 100 µM PFOS, which was significantly attenuated by PYP pretreatment (1–2 µg/mL) for 24 h prior to PFOS exposure, and the protective effect of the PYP treatment (1 µg/mL) was abolished by inhibiting the TrkB receptor antagonist with 200 nM of cyclotraxin B (Figure 1B,C). Thus, 100 µM PFOS and 1 µg/mL PYP were used to investigate the mechanism underlying the protective effects of PYP against PFOS-induced ER stress in vitro and in vivo.

### 2.2. PYP Pretreatment Downregulated PFOS-Induced Increase in GRP78 Expression and Intracellular Calcium Levels in Frontal Cortical Neurons

**Figure 2:**
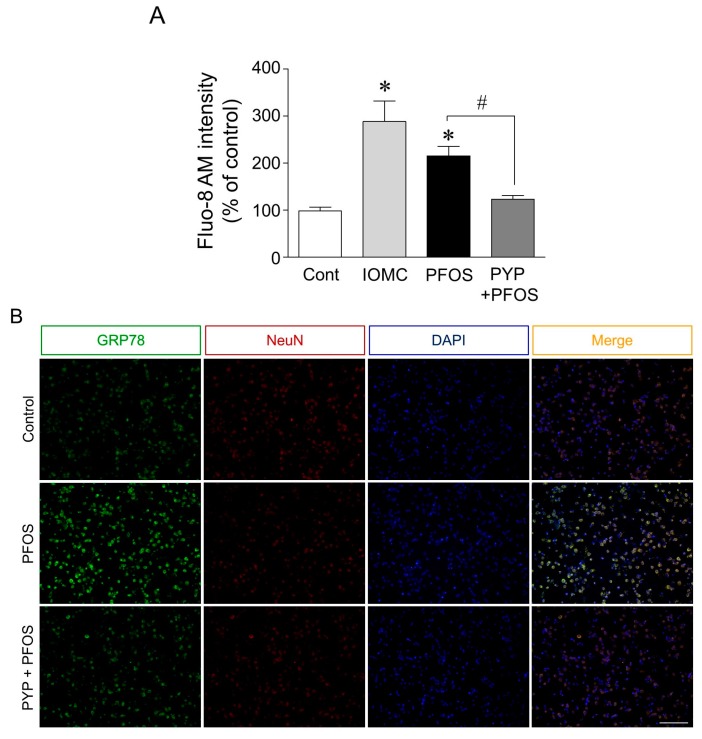
Effect of PYP on PFOS-induced intracellular calcium level and GRP78 expression. Cells were treated with 100 µM PFOS for 24 h, with or without PYP pretreatment (1 µg/mL) for 24 h prior to PFOS exposure. Fluo-8 AM intensity and GRP78 immunofluorescence were determined. Intracellular calcium levels detected by Fluo-8 AM significantly increased due to PFOS exposure for 24 h, which was attenuated by PYP (1 µg/mL) pretreatment. The ionomycin (1 µM) treated group was used as the positive control (**A**); The exposure to PFOS significantly upregulated GRP78 immunofluorescence in rat frontal cortical neurons, but the PYP pretreatment prior to PFOS exposure attenuated the PFOS-induced increase in GRP78 expression (**B**). The data were expressed as the mean ± SEM of three independent experiments, each performed in triplicate. * *p* < 0.05 versus control group; ^#^ *p* < 0.05 versus PFOS treatment; IOMC, ionomycin; Cont, control; NeuN, neuronal nuclear antigen. Scale bar represents 100 μm.

Since PYP pretreatment attenuated the decrease in frontal cortical neuron viability caused by PFOS exposure, we further investigated the role of PYP in altering cell viability and intracellular calcium levels, and regulating ER stress. Calcium levels in cortical neurons were determined by Fluo-8 AM intensity, using the ionomycin (1 µM) treated group as the positive control. ER stress in the frontal cortical neurons was assessed by analyzing the double-immunofluorescence levels of GRP78 and neuronal nuclear antigen (NeuN) proteins. As shown in Figure 2A, the intracellular calcium level was significantly increased by PFOS (100 µM) after 24 h, which was attenuated by PYP pretreatment for 24 h prior to PFOS exposure. The PFOS exposure also increased the immunofluorescence level of GRP78 in the cortical neurons, which was also downregulated by the PYP (1 µM) pretreatment prior to PFOS exposure (Figure 2B).

### 2.3. PFOS-Induced GRP78 Expression Was Mediated by Phosphorylation of JNK Linked to CaMKII and PYP-Mediated Decrease in GRP78 Expression Was Carried Out by Activating TrkB Receptor-PI3K-ERK1/2 Signaling

**Figure 3:**
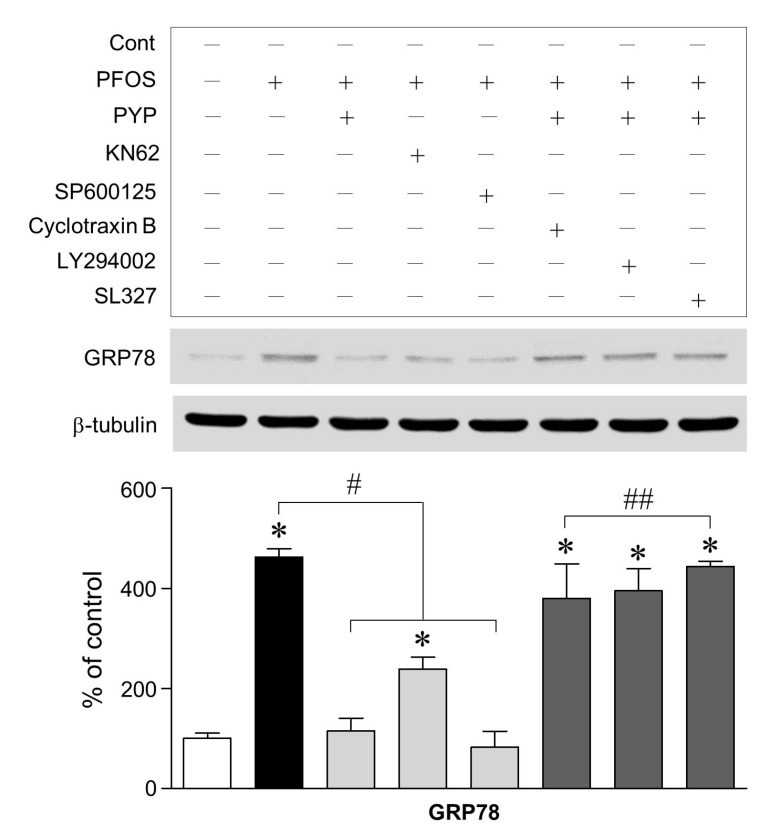
Effect of PYP pretreatment on PFOS-induced GRP78 expression and its underlying mechanisms. Inhibition of CaMKII and JNK phosphorylation with KN62 (10 µM) and SP600125 (10 µM), respectively, decreased the PFOS-induced increase in GRP78 expression as effectively as the PYP (1 µg/mL) pretreatment. The PYP-induced decrease in GRP78 expression was abolished by blocking the TrkB receptor and inhibiting PI3K and ERK1/2 activation with cyclotraxin B (200 nM), LY294002 (20 µM), and SL327 (10 µM), respectively. The data were expressed as the mean ± SEM of three independent experiments, each performed in triplicate. * *p* < 0.05 versus control group; ^#^ *p* < 0.05 versus PFOS treatment; ^##^ *p* < 0.05 versus PYP pretreatment; Cont, control.

To establish the mechanism underlying the PYP-induced decrease in GRP78 expression, we determined whether: (1) phosphorylation of calcium/calmodulin-dependent protein kinase II (CaMKII) and c-Jun N-terminal kinase (JNK) was associated with PFOS-induced ER stress, and (2) CaMKII/JNK-mediated ER stress was regulated by activation of PYP-induced TrkB receptor-PI3K-extracellular signal-regulated kinases 1/2 (ERK1/2) signaling. As shown in Figure 3, the inhibition of CaMKII and JNK phosphorylation with specific inhibitors for CaMKII and JNK, KN62 (10 µM) and SP600125 (10 µM), respectively, attenuated the PFOS-induced increase in GRP78 expression in frontal cortical neurons. Furthermore, blockade of TrkB receptor with cyclotraxin B (200 nM) and inhibition of PI3K and ERK1/2 with inhibitors for PI3K and ERK1/2, LY294002 (20 µM) and SL327 (10 µM), respectively, 30 min prior to PYP pretreatment, significantly abolished the PYP-induced decrease in GRP78 expression caused by PFOS exposure.

### 2.4. PYP Pretreatment Downregulated the Increase in PFOS-Induced CaMKII and JNK Phosphorylation and Inhibition of CaMKII Attenuated the Increase in PFOS-Induced JNK Phosphorylation in Frontal Cortical Neurons

**Figure 4:**
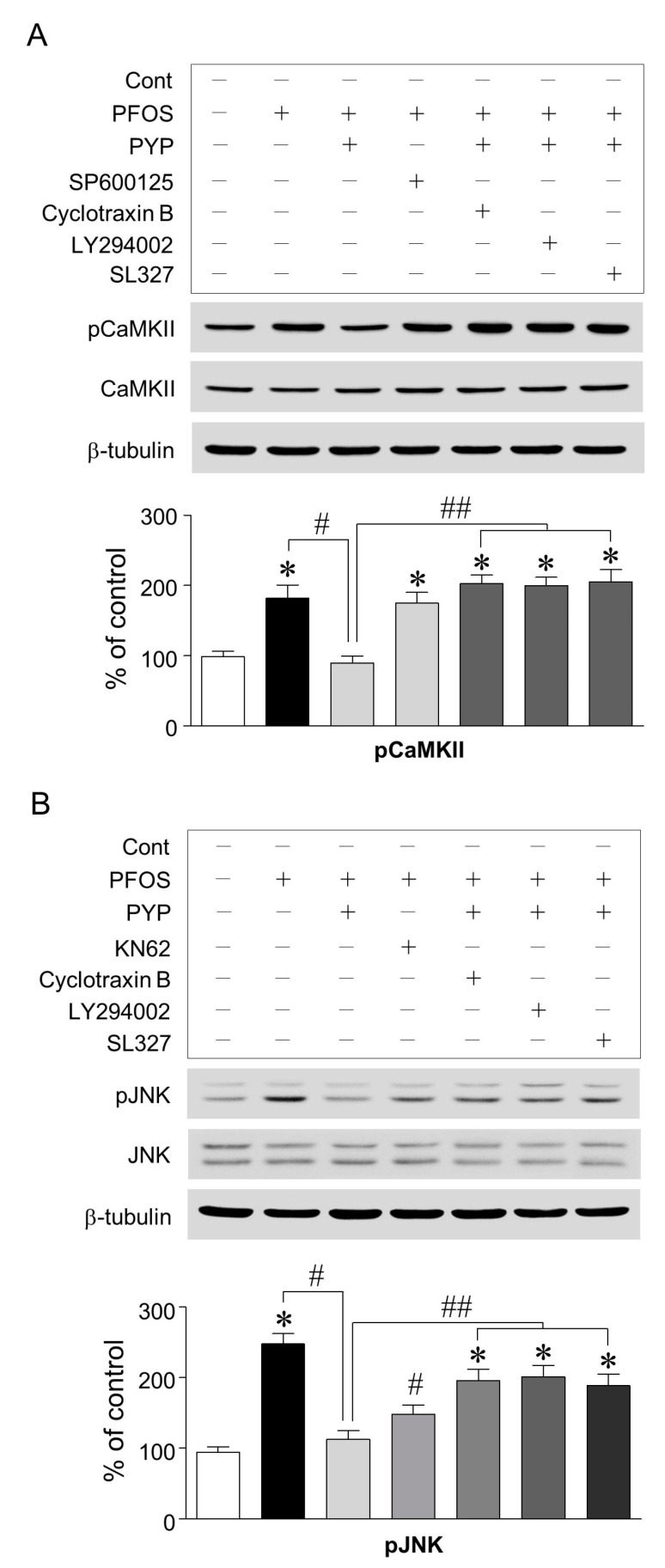
PYP-induced decrease in phosphorylation of JNK linked to CaMKII by PFOS exposure. CaMKII phosphorylation was upregulated due to PFOS exposure and downregulated by PYP (1 µg/mL) pretreatment. The PYP-induced decrease in CaMKII phosphorylation was abolished by blocking the TrkB receptor and inhibiting PI3K and ERK1/2 activation with cyclotraxin B (200 nM), LY294002 (20 µM), and SL327 (10 µM), respectively. Inhibiting JNK did not affect the PFOS-induced increase in CaMKII phosphorylation (**A**); JNK phosphorylation was also downregulated by PYP-induced TrkB receptor-linked ERK1/2 activation. The PFOS-induced increase in JNK phosphorylation was particularly downregulated by inhibiting CaMKII activation with 10 µM of KN62 (**B**). The data were expressed as the mean ± SEM of three independent experiments, each performed in triplicate. * *p* < 0.05 versus control group; ^#^ *p* < 0.05 versus PFOS treatment; ^##^ *p* < 0.05 versus PYP pretreatment; Cont, control.

Since the inhibition of CaMKII and JNK attenuated the increase in PFOS-induced GRP78 expression in frontal cortical neurons, we further investigated if PYP downregulated CaMKII and JNK phosphorylation and if there was any interaction between CaMKII and JNK. PFOS (100 µM) exposure significantly increased CaMKII phosphorylation in frontal cortical neurons, which was downregulated by PYP pretreatment (1 µg/mL). The decrease in PFOS-induced CaMKII phosphorylation by PYP pretreatment was abolished by blocking the TrkB receptor and inhibiting PI3K and ERK1/2 activation with cyclotraxin B (200 nM), LY294002 (20 µM), and SL327 (10 µM), respectively, 30 min prior to PYP pretreatment (Figure 4A). Similarly, increase in JNK phosphorylation via PFOS exposure was also attenuated by PYP pretreatment, which was also significantly downregulated by blocking the TrkB receptor and inhibiting PI3K and ERK1/2 activation (Figure 4B). Additionally, inhibiting CaMKII activation with KN62 (10 µM) downregulated the increase in PFOS-induced JNK phosphorylation; however, JNK inhibition with SP600125 (10 µM) did not affect PFOS-induced CaMKII phosphorylation. No significant difference was observed in total CaMKII and JNK expression when compared to the control.

### 2.5. Microinjection of PYP Downregulated the Increase in PFOS-Induced ER Stress in Rat Prefrontal Cortex

**Figure 5:**
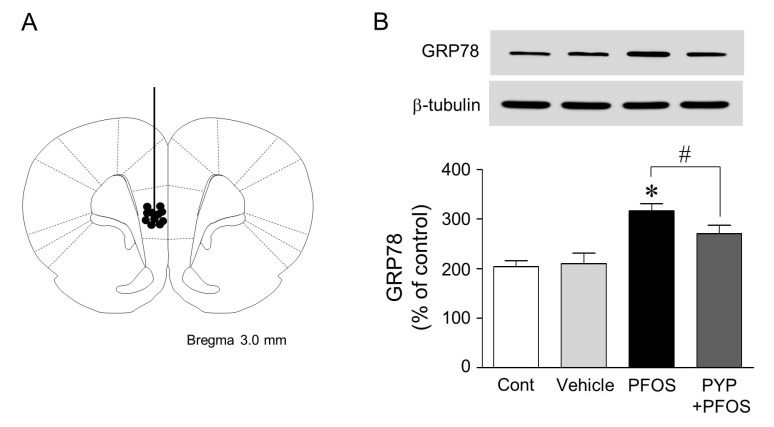
Effect of PYP treatment on PFOS-induced ER stress in rat prefrontal cortex. A schematic diagram showing the location of the internal cannulae tips (circles) that were located within the rat medial prefrontal cortex. Section +3.0 mm from the bregma (**A**); PFOS (10 mg/kg) injections once a day for 2 weeks increased the expression of GRP78 in the medial prefrontal cortex, which was reduced by PYP (1 µg/kg, 0.54 nmol) infusion to the prefrontal cortex 24 h before PFOS exposure (*n* = 4–6 per group) (**B**). * *p* < 0.05 versus control group; ^#^ *p* < 0.05 versus PFOS injection; Cont, control.

Finally, since PYP treatment downregulated PFOS-induced ER stress in frontal cortical neurons, we investigated if PYP protected neurons in rat prefrontal cortex from ER stress caused by PFOS exposure. The PFOS (10 mg/kg) dose was determined according to a previous study [27]. Rats were injected intraperitoneally with 10 mg/kg of PFOS dissolved in DMSO once a day for 2 weeks. A minimal concentration of DMSO (<0.01%) was used as a vehicle to prevent cellular damage. As shown in Figure 5, PFOS exposure for 2 weeks increased GRP78 expression in rat medial prefrontal cortex, which was reduced by the direct infusion of PYP (1 µg/kg, 0.54 nmol) to the cortex 24 h prior to PFOS exposure.

## 3. Discussion

PFOS, considered as a persistent organic pollutant, can accumulate in the brain and induce oxidative stress, resulting in ER stress and neurotoxicity. This study aimed to investigate the protective role of PYP against PFOS-induced ER stress in frontal cortical neurons and the mechanisms underlying its neuroprotective effects. In rat frontal cortical neurons, PYP pretreatment enhanced cell viability by downregulating the PFOS-induced GRP78 expression. The increase of PFOS-induced GRP78 expression was mediated by the phosphorylation of JNK linked to CaMKII, which also was downregulated by PYP pretreatment. The PYP-induced decrease in GRP78 expression by PFOS was abolished by blocking the TrkB receptor and inhibiting PI3K and ERK1/2 activation. These findings indicated that the neuroprotective effect of PYP was associated with the inhibition of JNK phosphorylation linked to CaMKII via the activation of TrkB receptor-PI3K-ERK1/2 signaling. Furthermore, the direct infusion of PYP to rat prefrontal cortex reduced PFOS-induced ER stress. The data suggest that PYP regulated the ER stress in frontal cortical neurons caused by the PFOS-induced perturbation of calcium homeostasis.

Many studies have explored the functional effects of peptides from *P. yezoensis*, which has exhibited enhanced cell viability by reducing ER stress induced via environmental pollutants [28]. It also had the ability to decrease oxidative stress caused by hydrogen peroxide and acetaminophen in Chang cells [21,23,24]. A recent study also demonstrated that a *P. yezoensis* peptide attenuated muscle atrophy in murine C2C12 myoblasts [26]. PFOS adversely affects the central nervous system because of its ability to cross the blood-brain barrier, resulting in neuroendocrine dysregulation, cognitive deficit, and developmental delay of neonatal growth [9,10,11,13,27]. PFOS particularly causes dysregulation of calcium-dependent signaling by disturbing the calcium homeostasis in the brain, which is mainly associated with ER stress. This data suggests that a functional peptide from *P. yezoensis* could contribute to maintaining healthy brain function against ER stress caused by persistent organic pollutants.

In this study, acute PFOS (100 µM) exposure for 24 h decreased frontal cortical neuron cell viability (Figure 1A). This decrease in cell viability was downregulated by PYP (1 µg/mL) pretreatment 24 h prior to PFOS exposure, and could be prevented by inhibiting the TrkB receptor with 200 nM of cyclotraxin B (Figure 1B,C). These results demonstrated that PYP could protect the frontal cortical neurons against PFOS exposure. Moreover, immunofluorescence levels of GRP78 expression in frontal cortical neurons were increased by PFOS exposure, which was downregulated by PYP pretreatment (Figure 2A). Similarly, PFOS-induced intracellular calcium levels in the cortical neurons were also downregulated by PYP pretreatment (Figure 2B). A previous study demonstrated that PFOS exposure elevated intracellular calcium concentration in hippocampal neurons followed by an increase in oxidative stress [29]. This data suggests that PYP-enhanced cell viability against PFOS exposure could be associated with the regulation of ER stress linked to calcium disturbance in frontal cortical neurons.

Increase in PFOS-induced GRP78 expression was downregulated by PYP pretreatment or inhibition of CaMKII and JNK. PYP-mediated decrease of ER stress was abolished by blocking the TrkB receptor and inhibiting PI3K and ERK1/2 activation with cyclotraxin B (200 nM), LY294002 (20 µM), and SL327 (10 µM), respectively, 30 min prior to PYP pretreatment (Figure 3). These findings demonstrated that PFOS-induced GRP78 expression was mediated by CaMKII and JNK phosphorylation. The activation of TrkB receptor-linked ERK1/2 signaling was also necessary for PYP-induced regulation of ER stress against PFOS exposure. This data suggests that the regulation of the PFOS-induced GRP78 expression by PYP could be associated with the phosphorylation of CaMKII and JNK.

As shown in Figure 4, the PFOS-induced increase in CaMKII and JNK phosphorylation was downregulated by PYP pretreatment, which was abolished by blocking the TrkB receptor and inhibiting PI3K and ERK1/2 activation with cyclotraxin B (200 nM), LY294002 (20 µM), and SL327 (10 µM), respectively. JNK phosphorylation by PFOS was particularly downregulated by the inhibition of CaMKII activation. These findings indicated that PYP decreased PFOS-induced ER stress by inhibiting the phosphorylation of JNK linked to CaMKII via activation of TrkB-linked ERK1/2 signaling in frontal cortical neurons. JNK plays a critical role in neuronal death via external stresses. Kainic acid-induced excitotoxicity and ischemia-induced cell death are both mediated by the activation of JNK signaling [30,31]. In neurodegenerative models, JNK induces in vitro apoptosis via the Alzheimer’s disease-related protein, β-amyloid, and is associated with dopaminergic neuronal death in the substantia nigra pars compacta of rats injected with 6-hydroxydopamine, which causes the death of dopaminergic neurons [32,33]. The neurotoxin, 1-methyl-4-phenyl-1,2,3,6-tetrahydropyridine, which induces dopaminergic neuronal loss resulting in Parkinson’s disease, is also regulated by JNK signaling in vivo [34]. These results suggest that regulating JNK phosphorylation via PYP-induced ERK1/2 activation results in the neuroprotective effects of PYP.

Finally, we investigated the protective effect of PYP in rat prefrontal cortex. The prefrontal cortex plays a key role in depression, anxiety, stress, and in controlling goal-directed behavior through extensive connections with other regions of the brain [35,36,37]. Thus, the increase in PFOS-induced ER stress in rat prefrontal cortex manifests as severe behavioral disorders. In this study, the intraperitoneal injection of PFOS (10 mg/kg) for 2 weeks, resulting in neuroendocrine dysregulation, increased ER stress in rat medial prefrontal cortex. Consecutive microinjections of PYP (1 µg/kg, 0.54 nmol) 24 h prior to PFOS exposure slightly downregulated PFOS-induced GRP78 expression in the prefrontal cortex (Figure 5). Many studies have correlated the dysregulation of ER stress with cognitive impairments and neurodegenerative disorders such as Alzheimer’s, Parkinson’s, and Huntington’s diseases [38,39]. Recent studies demonstrated that PFOS damaged synaptic plasticity by decreasing GluR2 expression and induced tau hyperphosphorylation, which are involved in PFOS-induced neurotoxicity [14,20,40]. These results indicated that PYP treatment downregulates ER stress in rat prefrontal cortex caused by PFOS exposure, suggesting that PYP could protect the cortical neurons from ER stress-induced neuronal dysfunction.

## 4. Materials and Methods

### 4.1. Peptide and Chemicals

PYP (YVSYALLAGDPSVLEDR; GenBank accession No. YP_537048) derived from phycoerythrin of *P. yezoensis* was identified using two-dimensional gel electrophoresis and synthesized by Peptron Inc. (Daejeon, Korea). Briefly, frozen samples of *P. yezoensis* (1 g) were mixed with 50 mL of 0.1 M sodium acetate buffer (pH 6.0) for 16 h with continuous shaking at 4 °C and filtered through Whatman filter paper. This was then further precipitated by methanol and chloroform [41]. The pellet was purified with the 2-D Clean-up Kit (GE Healthcare, Princeton, NJ, USA) followed by isoelectric focusing and SDS-PAGE according to the manufacturer’s protocols (GE Healthcare). Based on a previous study [42], the protein of interest was excised and digested with trypsin, desalted using a constricted GELoader^®^ tip (Eppendorf, Hamburg, Germany) packed with a custom-made chromatographic column consisting of 100–300 nL of POROS reverse phase R2 material (20–30 µm bead size; PerSeptive Biosystems, Framingham, MA, USA), and followed by nano-ESI using a MicroQ-TOF III mass spectrometer (Bruker Daltonics, Bremen, Germany). The source temperature was room temperature and a potential of 1 kV was applied to the pre-coated borosilicate nanoelectrospray needles (EconoTip™, Woburn, MA, USA) in the ion source combined with a nitrogen back-pressure of 0–5 psi to produce a stable flow rate (10–30 nL/min). The cone voltage was 800 V. The quadrupole analyzer was used to select precursor ions for fragmentation in the hexapole collision cell. The collision gas Ar was applied at a pressure of 6–7 × 10^−5^ mbar and the collision energy was 15–40 V. Product ions were analyzed using an orthogonal TOF analyzer fitted with a reflector, a micro-channel plate detector, and a time-to-digital converter. The data was processed using a peptide sequence system. The generated peptide fragment files were used to query either the SwissProt or NCBI database using the MASCOT software (Matrix Science, Boston, MA, USA).

To understand their function in the brain, the identified peptide was newly synthesized by Peptron. Peptide purification was performed on a CAPCELL PAK C18 column (Shiseido, Tokyo, Japan) attached to a high-performance liquid chromatography apparatus with elution in 0.1% trifluoroacetic acid under a 3–70% acetonitrile gradient at a flow rate of 1 mL/min with UV detection at 220 nm.

PFOS (CAS 2795-39-3, >98%) and dimethyl sulfoxide (DMSO; CAS 67-68-5, >99.9%) were purchased from Sigma-Aldrich (St. Louis, MO, USA). JNK inhibitor (SP600125; 10 µM), CaMKII inhibitor (KN62; 10 µM), TrkB receptor antagonist (cyclotraxin B; 200 nM), PI3K inhibitor (LY294002; 20 µM), and ERK1/2 inhibitor (SL327; 10 µM) were obtained from Tocris Bioscience (Minneapolis, MN, USA) and were incubated for 30 min prior to PYP or PFOS treatments.

### 4.2. Primary Cortical Neuron Culture

Primary cortical neurons were prepared from E18 rat embryos based on a previous study [43]. All animal use procedures were approved by the Animal Ethics Committee of Pukyong National University (Approval No. 2017-24) and carried out in accordance with the guidelines for the care and use of laboratory animals. Briefly, pregnant rats were deeply anesthetized with a mixture of Zoletil 50 (18.75 mg/kg; Virbac Korea, Seoul, Korea) and Rompun (5.83 mg/kg; Bayer Korea, Seoul, Korea), and the uteri were dissected out. The frontal cortex from the intact brains was dissected out into calcium-, magnesium-, and bicarbonate-free Hank’s balanced salt solution buffered (Gibco, Grand Island, NY, USA) with 10 mM HEPES (pH 7.3). After incubation with trypsin for 15 min at 37 °C, the frontal cortex was dissociated by repeated up and down pipetting using a narrow tip. The cells were then plated onto poly-d-lysine-coated 6-well plates at a density of 2 × 10^5^ cells/well and maintained in Neurobasal medium supplemented with B27 (Gibco) at 37 °C in a humidified incubator containing 5% CO_2_. After 24 h of incubation, cytosine arabinoside, at a final concentration of 5 µM, was added to inhibit glial proliferation. Half of the medium was changed every third day, and the cultures were used for experiments 12–14 days after plating.

### 4.3. Cell Viability Assay

Cell viability was determined by Cyto X (LPS Solution, Daejeon, Korea). Cells were seeded at 2 × 10^4^ cells per well in a 96-well plate containing a final volume of 100 µL per well and incubated for 24 h at 37 °C in a humidified incubator containing 5% CO_2_. Following exposure to PFOS (25–400 µM) for 24 h with or without PYP pretreatments (0.25–2 µg/mL) for 24 h, a water-soluble tetrazolium salt (10 µL/well) was added and incubated for 60 min at 37 °C in a 5% CO_2_ incubator. All the antagonists or inhibitors were applied for 30 min before the PYP treatment. Colored formazan was measured by examining the absorbance at 450 nm.

### 4.4. Double-Immunofluorescence Staining

Double-immunostaining was performed to explore the expression of GRP78 in rat cortical neurons. Following two washes with DPBS containing Ca^2+^ and Mg^2+^, cultured neurons were fixed with 4% paraformaldehyde for 20 min and permeabilized with 0.3% Triton X-100 (diluted in DPBS containing Ca^2+^ and Mg^2+^) for 5 min at room temperature. After three washes with PBS, the cells were incubated for 60 min at room temperature with a 5% goat serum solution in DPBS containing Ca^2+^ and Mg^2+^, and then overnight at 4 °C in a mixture of rabbit anti-GRP78 and mouse anti-NeuN primary antibodies diluted at 1:500. After washing thrice with DPBS containing Ca^2+^ and Mg^2+^, the cells were incubated in a mixture of two secondary antibodies (goat anti-rabbit IgG-Alexa Fluor 488 and goat anti-mouse IgG-Alexa Fluor 647) at a dilution of 1:500 for 60 min at room temperature. Following three washes and staining with DAPI solution for 10 min, the cells were mounted with a drop of Prolong gold anti-fade reagent (Gibco). Antibodies and normal goat serum for double-immunostaining were purchased from Abcam (Cambridge, UK). The fluorescent images were taken using the EVOS^®^ FL Cell Imaging System (ThermoFisher Scientific, Waltham, MA, USA).

### 4.5. Immunoblotting

Cortical neurons were seeded at a density of 2 × 10^5^ cells/well in a 6-well plate and treated with PFOS (100 µM) for 24 h with or without PYP (1 µg/mL) pretreatment. Following two washes with PBS, frontal cortical neuron lysis was performed in RIPA buffer containing a protease inhibitor cocktail (Cell Signaling Technology, Danvers, MA, USA). Protein concentrations were determined using a BCA protein assay kit (ThermoFisher Scientific), and proteins (20 µg) were separated using 12–15% SDS-polyacrylamide gel electrophoresis. Separated proteins were transferred to a polyvinylidene difluoride membrane. The membrane was blocked with a blocking buffer containing 1% BSA in Tris-buffered saline and 0.1% Tween-20 (TBST), and then probed overnight at 4 °C on a shaker with primary antisera for GRP78, JNK, phospho-JNK, CaMKII, phospho-CaMKII, and β-tubulin (Cell Signaling Technology), each at a dilution of 1:1000. After washing thrice with TBST for 10 min, the membranes were incubated with a corresponding secondary antiserum (ThermoFisher Scientific) at a dilution of 1:10,000 for 60 min at room temperature. The membrane was stripped and reprobed with anti-β-tubulin antibody to normalize the blots.

### 4.6. Intracellular Calcium Level

Cortical neurons were seeded at a density of 2 × 10^4^ cells/well in a 96-well plate. On Day 12, the cells were incubated with 4 µM Fluo-8 AM (Abcam) in neuronal medium for 1 h with or without PYP pretreatments (1 µg/mL) for 24 h, and then exposed to 100 µM PFOS for 24 h at 37 °C in a 5% CO_2_ incubator. After washing twice with calcium-, magnesium-, and bicarbonate-free Hank’s balanced salt solution buffer with 10 mM HEPES (pH 7.3), the fluorescence changes were measured at excitation (480 nm) and emission wavelengths (535 nm). The ionomycin (1 µM) treated group was used as positive control.

### 4.7. Rat Surgery and Microinjection

Rats were deeply anesthetized with a mixture of Zoletil 50 (18.75 mg/kg; Virbac Korea) and Rompun (5.83 mg/kg; Bayer Korea) and placed in a stereotaxic apparatus (Stoelting Co., Wood Dale, IL, USA). Under aseptic conditions, infusion guide cannula (22 gauge; Plastics One, Roanoke, VA, USA) was implanted into the cingulate cortex using the following coordinates from the bregma: anterior-posterior, +3 mm; dorsal-ventral, −4 mm; medial-lateral, 0.5 mm. After a 28-gauge dummy cannula was inserted to prevent the guide cannula from clogging, the rats were given at least 3 days to recover. Following replacement with a 28-gauge internal cannula that protruded 0.5 mm beyond the guide cannula, PYP (1 µg/kg, 0.54 nmol) or artificial cerebrospinal fluid was infused into the right medial prefrontal cortex using a 0.2 µL Hamilton microsyringe (Reno, NV, USA) in freely moving rats, 24 h prior to PFOS (10 mg/kg) that was injected intraperitoneally once a day for 2 weeks based on a previous study [27]. After PYP microinjection, the internal cannula was left in place for an additional 5 min to prevent any possible backflow.

### 4.8. Statistics

Data is represented as mean ± SEM for each group (*n* = 4–6). Statistical significance between the groups was determined by one-way ANOVA with repeated measures followed by Tukey’s post-hoc test using the GraphPad software (Prism 5, La Jolla, CA, USA). *p* < 0.05 was considered to be statistically significant.

## 5. Conclusions

**Figure 6:**
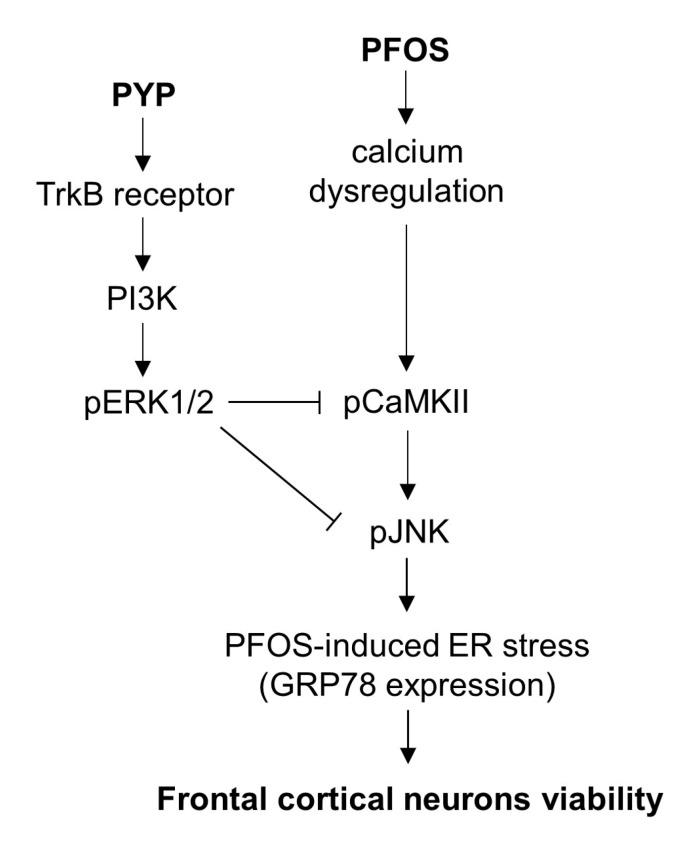
Schematic of a proposed mechanism underlying the neuroprotective effects of PYP against PFOS exposure in frontal cortical neurons. The expression level of GRP78 by PFOS exposure is mediated by phosphorylation of JNK linked to CaMKII. PYP downregulates the JNK-mediated increase in ER stress by PFOS via the activation of TrkB receptor-linked ERK1/2 signaling. Thus, PYP protects frontal cortical neurons from ER stress caused by PFOS-induced calcium dysregulation.

Collectively, these findings demonstrate that PYP enhances frontal cortical neuron viability via the activation of TrkB receptor-ERK1/2 signaling (Figure 6) and attenuation of ER stress in rat prefrontal cortex against PFOS exposure. The data also suggests that PYP reduces the PFOS-mediated susceptibility to calcium dysregulation, possibly alleviating the cognitive deficits and behavioral disorders associated with persistent organic pollutants.
